# Photobiomodulation therapy in keloid management: a comprehensive review

**DOI:** 10.3389/fmed.2025.1550662

**Published:** 2025-07-09

**Authors:** Arya Tjipta Prananda, Rony Abdi Syahputra

**Affiliations:** ^1^Faculty of Medicine, Universitas Sumatera Utara, Medan, Indonesia; ^2^Department of Pharmacology, Faculty of Pharmacy, Universitas Sumatera Utara, Medan, Indonesia

**Keywords:** keloid, photobiomodulation therapy, low-level laser therapy, fibroblast, TGF-β1, collagen synthesis, wound healing, scar management

## Abstract

Keloid formation is a pathological scarring process marked by excessive fibroblast activity, overproduction of extracellular matrix (ECM), and chronic inflammation, presenting significant challenges in management despite existing treatments like corticosteroid injections, surgical excision, and cryotherapy. This review evaluates Photobiomodulation Therapy (PBMT) as a promising non-invasive approach for keloid treatment. PBMT utilizes non-thermal light in the red to near-infrared spectrum, which enhances mitochondrial activity, reduces reactive oxygen species (ROS), and regulates fibroblast proliferation and apoptosis. It also exhibits anti-fibrotic properties by inhibiting TGF-β1 expression, collagen synthesis, and Smad signaling, while modulating inflammation through reduced pro-inflammatory cytokines (IL-6, TNF-α) and enhanced macrophage activity. Preclinical evidence in animal models and fibroblast cultures demonstrates PBMT’s ability to reduce scar size, collagen deposition, and fibroblast activity. Clinical studies, including randomized controlled trials (RCTs) and case reports, show significant improvements in keloid height, elasticity, and texture, with reductions in pain and pruritus, as well as lower recurrence rates compared to conventional therapies. PBMT is well-tolerated with minimal adverse effects, such as transient redness or mild itching, and is safe for all skin types, including those with darker pigmentation. In conclusion, PBMT offers a promising, safe, and effective alternative for keloid management by targeting key fibrotic, inflammatory, and angiogenic processes. However, further large-scale randomized controlled trials with standardized protocols are necessary to confirm its long-term efficacy and integrate it into clinical practice.

## Introduction

Keloids are pathological scars resulting from dysregulated wound healing, marked by excessive deposition of fibrous tissue that extends beyond the original wound margins (1, 2). In contrast to hypertrophic scars, keloids exhibit continuous growth in the absence of additional trauma (3). Clinically, keloids may present with pain, pruritus, and stiffness, potentially limiting mobility when located near joints, and contributing to psychological distress, particularly when situated in visible areas (4). They are more prevalent among individuals with darker skin phototypes and those with a genetic predisposition, typically developing between the ages of 10 and 30 years (5–9). As such, keloids constitute both cosmetic and clinical challenges.

The pathophysiology of keloids involves disrupted wound healing, particularly during the proliferation and remodeling phases, leading to excessive extracellular matrix (ECM) production—mainly type I and III collagen—and thick, irregular scars (10, 11). A key factor is fibroblast hyperactivity, resulting in increased synthesis of ECM components such as collagen, fibronectin, and proteoglycans (12). In keloid tissue, both fibroblasts and myofibroblasts are present and contribute to pathological scarring. Myofibroblasts typically arise through TGF-β1–induced differentiation of fibroblasts during wound healing, although contributions from other precursor cells—including pericytes and mesenchymal stem cells—have been proposed. In normal wound healing, myofibroblasts appear transiently to facilitate wound contraction and then undergo apoptosis. In keloids, this resolution phase is impaired, leading to persistent myofibroblast presence and continuous ECM deposition (13, 14). Vascular Endothelial Growth Factor (VEGF) supports angiogenesis, aiding keloid growth (15, 16), while chronic inflammation and cytokines like IL-6, IL-8, and TNF-α exacerbate the process.

Keloid treatment remains clinically challenging due to limited efficacy and high recurrence rates. Intralesional corticosteroids, particularly triamcinolone acetonide, are widely used to inhibit fibroblast proliferation and collagen synthesis but are associated with adverse effects such as skin atrophy, hypopigmentation, pain, and frequent recurrence (17–19). Surgical excision alone results in recurrence rates of 50–80% due to post-operative fibroblast activation and collagen overproduction (20). Cryotherapy shows inconsistent results and often induces hypopigmentation, especially in darker skin types. Radiotherapy is effective adjunctively but poses long-term carcinogenic risks. Silicone gel therapy relies heavily on patient adherence, limiting its practicality. These limitations highlight the urgent need for safer and more effective therapeutic strategies.

**Figure 1:**
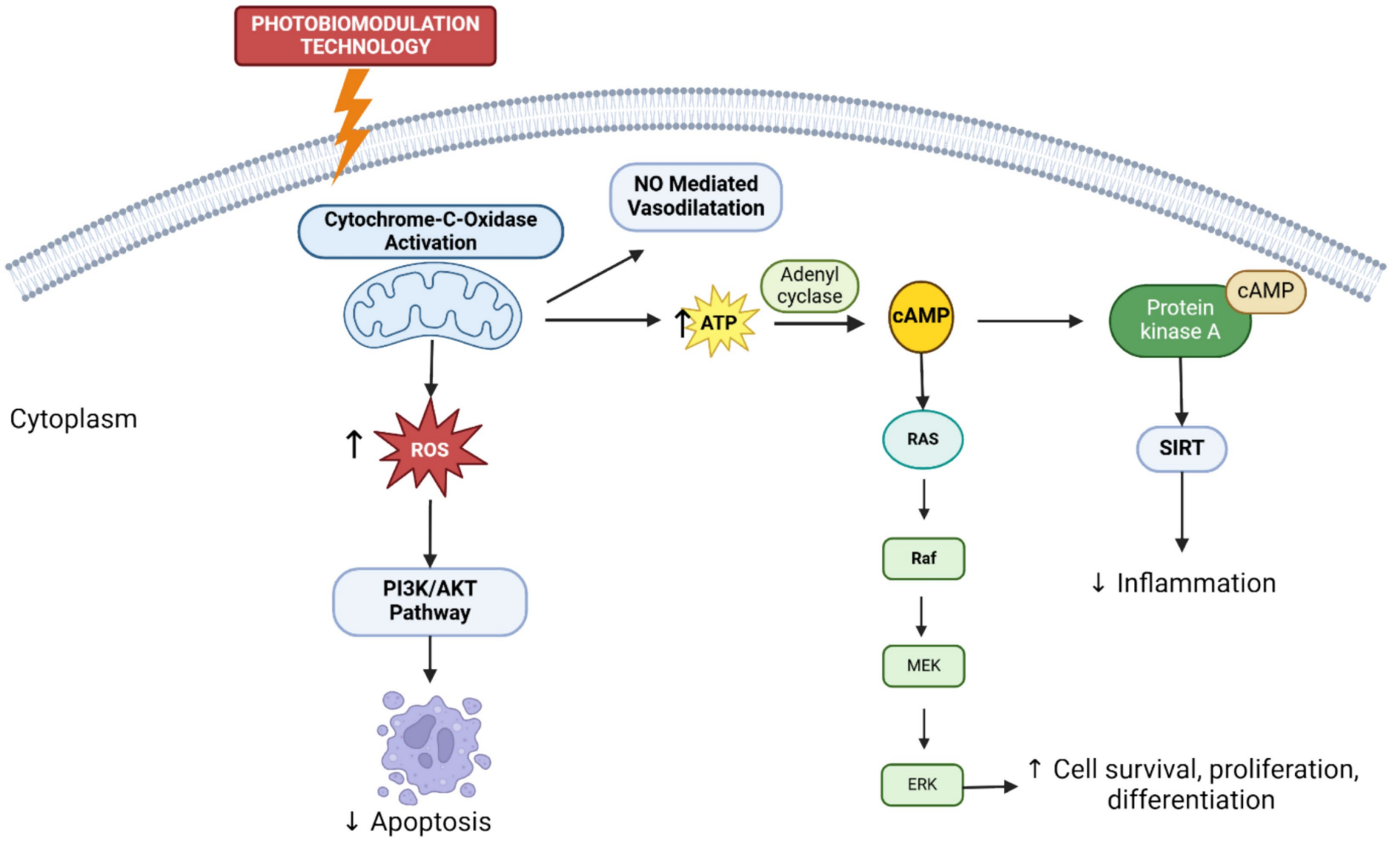
Mechanism of Photobiomodulation Therapy (PBMT). Photobiomodulation Therapy (PBMT) activates cytochrome c oxidase in mitochondria, boosting ATP and nitric oxide (NO) production, which promotes vasodilation. This also generates ROS, activating the PI3K/AKT pathway to support cell survival, proliferation, and differentiation. ROS and ATP-driven cAMP stimulate PKA, influencing the RAS–RAF–MEK–ERK cascade and regulating sirtuins (SIRT), which epigenetically modulate gene expression by deacetylating histones and transcription factors like NF-κB. This suppresses pro-inflammatory cytokines (e.g., TNF-α, IL-6), inhibits fibrosis, and improves mitochondrial function. Overall, PBMT enhances repair, metabolism, and regeneration through coordinated mitochondrial and signaling pathway activation.

In recent years, Photobiomodulation Therapy (PBMT), or Low-Level Laser Therapy (LLLT), is a non-thermal light-based treatment that uses low-energy lasers or LEDs to modulate cellular and molecular processes involved in keloid formation (21). Unlike tissue-destructive laser ablation, PBMT works by stimulating cellular activity through the absorption of light by intracellular chromophores, such as cytochrome c oxidase in mitochondria (Figure 1). By stimulating mitochondrial chromophores like cytochrome c oxidase, PBMT enhances ATP production and reduces reactive oxygen species (ROS), promoting cellular repair and balanced fibroblast activity (22). In keloid management, PBMT inhibits fibroblast proliferation, reduces collagen synthesis (23), and suppresses TGF-β1 expression (24). Its anti-inflammatory effects decrease IL-6 and TNF-α levels, while downregulation of VEGF disrupts angiogenesis, collectively limiting scar overgrowth.

PBMT offers several advantages over conventional therapeutic modalities, including its non-invasive nature, minimal risk of side effects, and versatility in combination with other treatments (25). PBMT can be used as a standalone or adjunctive therapy to enhance the efficacy of keloid management. Preclinical and clinical studies have demonstrated PBMT’s ability to significantly reduce keloid size, thickness, and hardness, improve tissue elasticity, and alleviate symptoms such as pain and itching (26, 27). Additionally, some studies report lower recurrence rates following PBMT treatment compared to other modalities. However, challenges remain, particularly regarding the variability of therapy protocols, including light wavelength, energy dose, session duration, and frequency. Standardizing PBMT parameters is crucial to ensure consistent and reproducible therapeutic outcomes across different populations.

This review provides a comprehensive analysis of the mechanisms underlying PBMT’s action, evaluates the scientific evidence from preclinical and clinical studies, and highlights future research directions. The focus is on understanding how PBMT modulates the cellular and molecular processes involved in keloid formation while critically assessing its clinical effectiveness and safety profile. By synthesizing current knowledge, this review aims to clarify PBMT’s potential role in keloid management and identify key areas for further investigation.

## Pathophysiology of keloid formation and the need for alternative therapeutics

### Normal wound healing

Wound healing is a dynamic, multi-phase process involving hemostasis, inflammation, proliferation, and remodeling, each essential for restoring tissue integrity (28). Hemostasis starts immediately after injury with platelet activation, coagulation, and fibrin clot formation to stop bleeding and support cell migration, during which platelets release growth factors like PDGF and TGF-β to recruit inflammatory cells and promote healing (29–31). The inflammatory phase begins within hours and lasts 2–3 days, during which neutrophils and macrophages clear debris and pathogens while releasing cytokines (IL-1β, IL-6, TNF-α) and growth factors to stimulate fibroblast activity and angiogenesis (32, 33). In the proliferation phase, fibroblasts produce ECM components such as collagen and fibronectin, epithelial cells migrate to cover the wound, and VEGF-driven angiogenesis ensures nutrient supply (34, 35). Remodeling involves the replacement of type III collagen with type I, mediated by MMPs, resulting in stronger scar tissue (36). Keloids result from dysregulation in the proliferation and remodeling phases, leading to abnormal ECM accumulation and scarring; hyperactive fibroblasts, stimulated by elevated levels of TGF-β1 and VEGF, drive excessive collagen synthesis—particularly types I and III—while reduced ECM degradation due to altered MMP activity and increased TIMPs promotes fibrotic tissue extending beyond the wound margin (37, 38), highlighting the complexity of keloid formation and its clinical challenges.

### Wound healing disorders in keloids

In keloids, dysregulated wound healing during the proliferation and remodeling phases is driven by fibroblast hyperactivity, excessive collagen I and III production, and imbalanced ECM turnover (39, 40). TGF-β1/β2 enhance fibroblast proliferation, differentiation of fibroblasts into myofibroblasts, and collagen synthesis while suppressing MMPs critical for ECM degradation (41). MMP regulation is complex—MMP1/MMP13 initiate collagen I breakdown, while low TIMP2 levels can activate MMP2. Broad MMP inhibition, such as targeting MMP9 in cancer, has failed due to adverse effects. VEGF promotes abnormal angiogenesis, sustaining fibrotic tissue, while IL-6 and IL-8 perpetuate inflammation and fibroblast activation. Myofibroblast resistance to apoptosis leads to ongoing collagen accumulation (42). Thus, ECM dysregulation in keloids involves both collagen overproduction and impaired degradation.

### Abnormal ECM remodeling in keloids

In keloids, ECM remodeling is profoundly impaired, leading to excessive collagen deposition and disorganized tissue architecture. Unlike the criss-cross collagen arrangement in healthy skin, keloids show thick, irregular, and haphazard collagen bundles due to reduced decorin and elevated collagen V, which disrupts fibril assembly. In healthy dermis, the dense and well-organized collagen matrix contributes to a biophysical phenomenon known as molecular crowding. This high-density environment restricts fibroblast proliferation by physically limiting cellular space and hindering the diffusion of signaling molecules, thus acting as a natural brake on overactivation. In contrast, artificial dermis in 3D skin models lacks such structural constraints, often allowing unregulated fibroblast activity. The normal replacement of type III with type I collagen during scar maturation is impaired, resulting in persistent type III and excessive type I collagen that form stiff, inflexible tissue (43). ECM imbalance is worsened by GAG overproduction, especially hyaluronic acid (HA), which binds water, increases osmotic pressure, expands ECM volume, and activates pro-fibrotic signaling via CD44. Notably, increased HA incorporation may reduce molecular crowding by hydrating and spacing the ECM, facilitating better diffusion of regulatory molecules and potentially modulating fibroblast activity. Other GAGs, like chondroitin sulfate, and fibronectin overproduction further stiffen the matrix and promote ECM persistence (37). Reduced decorin enhances TGF-β activity, while abnormal HA-CD44 signaling sustains fibroblast proliferation. Concurrently, decreased MMPs and elevated TIMPs inhibit ECM degradation, leading to keloids’ dense, rigid texture and invasive growth (44).

### Challenges in standard keloid treatments and the potential of PBMT as a therapeutic alternative

Keloid treatment remains challenging due to high recurrence rates and the lack of standardized protocols. Keloids often recur even after surgical excision, with recurrence rates reaching 50–80% without adjuvant therapies such as corticosteroids or radiotherapy (20, 45). Treatment outcomes vary with lesion site, size, patient age, and genetics, complicating management. The absence of consensus on optimal dosing, duration, or combination therapies highlights the need for more effective, minimally invasive approaches. Photobiomodulation therapy (PBMT) offers promise by enhancing mitochondrial function via cytochrome c oxidase activation, promoting ATP production and NO-mediated vasodilation, modulating redox balance, reducing inflammatory cytokines, and regulating PI3K/AKT and RAS–MEK–ERK pathways involved in fibroblast activity and ECM remodeling. PBMT may serve as a viable adjunct or alternative to current treatments, warranting further comparative studies on efficacy and safety.

## Working mechanism of photobiomodulation therapy

### Light absorption and cellular effects

PBMT uses 600–1,100 nm light (red to near-infrared) to non-invasively modulate cellular activity by enhancing mitochondrial function and ATP production through cytochrome c oxidase activation (46, 47). ATP supports key processes like proliferation, migration, and tissue repair. In keloids, PBMT regulates fibroblast activity, reduces ROS to prevent oxidative stress and collagen overproduction, and modulates the cell cycle to limit excessive ECM components such as type I and III collagen (48).

### Anti-fibrotic mechanisms in keloids

Photobiomodulation therapy (PBMT) demonstrates significant anti-fibrotic effects by modulating key pathophysiological mechanisms involved in keloid formation, including fibroblast activity, collagen synthesis, and pro-fibrotic signaling pathways. PBMT suppresses fibroblast proliferation by regulating intracellular pathways such as Akt/PI3K and ERK/MAPK, leading to G1 cell cycle arrest and apoptosis in hyperactive fibroblasts (49). It also downregulates the expression of collagen-encoding genes, such as COL1A1 and COL3A1, which code for type I and type III collagen respectively, and inhibits pro-fibrotic enzymes, thereby reducing collagen synthesis and preventing excessive extracellular matrix (ECM) accumulation (50). Additionally, PBMT attenuates the expression of transforming growth factor-beta 1 (TGF-β1), a central mediator in keloid pathogenesis, and inhibits its downstream Smad signaling pathway, further decreasing fibroblast activation and collagen production (51). Collectively, these mechanisms contribute to the restoration of ECM homeostasis and the attenuation of fibrotic progression.

### Immunomodulating effects

Chronic inflammation is a key factor in the initiation and persistence of keloid formation. PBMT exerts significant immunomodulatory effects by regulating the inflammatory response, thus fostering a wound healing microenvironment that minimizes excessive fibrosis. A primary mechanism is the reduction of pro-inflammatory cytokines, including IL-6, TNF-α, and IL-8, which stimulate fibroblast activity and collagen synthesis. By suppressing these cytokines, PBMT alleviates chronic inflammation that contributes to keloid pathogenesis. Additionally, PBMT promotes macrophage activation, particularly the transition to the M2 phenotype, which has anti-inflammatory and pro-regenerative properties. M2 macrophages aid in cellular debris clearance and the release of growth factors supporting wound healing (52, 53). This macrophage polarization also facilitates more balanced ECM remodeling, significantly reducing the risk of excessive ECM accumulation (54).

### Vascular effects

Excessive angiogenesis, driven by elevated VEGF expression, is a hallmark of keloid formation. PBMT regulates angiogenesis by suppressing VEGF expression and associated signaling pathways, thereby inhibiting the formation of new blood vessels in the wound area (55). This reduction in angiogenesis limits the nutrient supply required for fibroblast activity and ECM accumulation. Additionally, PBMT stabilizes existing blood vessel structures, reducing vascular leakage and preventing excessive blood supply to the wound, thus promoting balanced vascularization and supporting wound healing without exacerbating fibrous tissue growth (56).

### Summary of PBMT vs. other energy-based modalities

**Figure 2:**
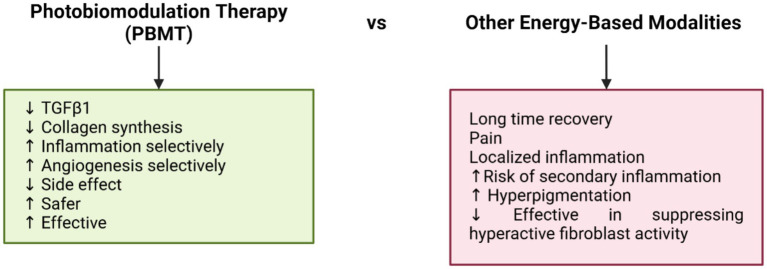
Photobiomodulation therapy advantages vs. other energy based modalities disadvantages.

PBMT offers several advantages over other energy-based therapies, such as laser ablation, fractional CO₂ laser, and intense pulsed light (IPL). Laser resurfacing, for instance, uses thermal energy to destroy surface tissue but often results in secondary inflammation, hyperpigmentation, and prolonged recovery (57). Fractional CO₂ lasers stimulate ECM remodeling through thermal micro-columns but are frequently associated with pain and localized inflammation (58). IPL targets pigmentation and vascularization but is less effective in suppressing fibroblast activity. In contrast, PBMT operates non-thermally and non-invasively, addressing cellular and molecular mechanisms of keloid pathogenesis. PBMT suppresses TGF-β1, reduces collagen synthesis, and modulates inflammation and angiogenesis, with minimal side effects (59). This makes PBMT a safer, more flexible, and effective option, either as a standalone treatment or in combination with other therapies (Figure 2). PBMT offers a distinct, non-invasive mechanism and is often combined with non-energy-based therapies for enhanced outcomes. Corticosteroids and 5-fluorouracil (5-FU), commonly used to suppress fibroblast activity, are effective but may cause adverse effects such as skin atrophy, hypopigmentation, pain, or ulceration. Agents like bleomycin and verapamil also target keloid fibroblasts but show variable efficacy and safety. PBMT can enhance these treatments by improving drug penetration, reducing inflammation, and supporting scar remodeling, making it a promising standalone or adjunct option, particularly for resistant keloids.

## Evidence from preclinical and clinical studies

### Preclinical studies

PBMT has shown efficacy in treating pathological scarring, including keloids. In animal models, it significantly reduced scar size and thickness; for example, 810 nm light inhibited fibroblast proliferation and collagen deposition in mice and rabbits (60). Histology revealed thinner scars with normalized collagen patterns, along with increased MMP activity for ECM remodeling (61). PBMT also reduced inflammation and improved tissue elasticity, indicating antifibrotic effects. *In vitro*, 660 nm and 830 nm PBMT suppressed keloid fibroblast proliferation by modulating the cell cycle and reducing S-phase cells (62, 63). It also downregulated TGF-β1, key in keloid pathogenesis (64), and induced fibroblast apoptosis via cytochrome c and caspase-3 activation (65). These findings support PBMT’s potential as a targeted therapy for keloids.

### Clinical studies

Clinical studies strongly support PBMT’s effectiveness in reducing keloid size, thickness, and symptoms. Evidence from RCTs, observational studies, and case reports aligns with pre-clinical findings. For example, PBMT using 810 nm at 4 J/cm^2^ for 12 weeks significantly reduced keloid height and improved skin tone (66). Other studies using 660 nm and 830 nm wavelengths also showed enhanced skin elasticity, reduced pain, and improved texture with lower recurrence risk (67, 68). These outcomes confirm PBMT’s role in inhibiting fibroblast overgrowth and promoting ECM remodeling.

PBMT is a safe, well-tolerated, non-invasive treatment with minimal, temporary side effects such as redness or warmth (69). It is especially suitable for darker skin types prone to post-inflammatory hyperpigmentation and scarring, as it modulates inflammation without damaging the epidermis (70). With minimal recovery time and a favorable safety profile, PBMT is a reliable alternative to invasive keloid therapies (71).

## Challenges and limitations of PBMT

Despite the promising potential of photobiomodulation therapy (PBMT) in keloid management, several challenges and limitations must be addressed to optimize its clinical implementation. A key issue is the variability in PBMT protocols, including differences in energy dose, light wavelength, session duration, and frequency, with studies reporting wavelengths between 600 and 1,100 nm, energy levels of 4–8 J/cm^2^, and varying session durations (72, 73), making comparisons difficult and hindering the establishment of optimal parameters. Standardizing these protocols through systematic research is essential. Furthermore, the lack of large-scale, multicenter clinical trials remains a significant limitation, as existing studies often involve small sample sizes, limited designs, and short follow-up periods, thus reducing the generalizability of findings. To support PBMT’s inclusion in clinical guidelines, large-scale randomized controlled trials (RCTs) with diverse populations are necessary. Additionally, individual variability in response to PBMT—shaped by factors such as genetics, scar location, size, skin type, and age (25)—complicates outcome prediction and underscores the need for personalized treatment strategies. Overall, addressing protocol inconsistencies, expanding clinical evidence through robust trials, and adopting personalized approaches will be crucial to enhance the efficacy, safety, and clinical adoption of PBMT for keloid treatment.

## Future perspectives and research directions

Photobiomodulation therapy (PBMT) shows strong potential as a primary treatment for keloids, though further research is needed to refine its clinical application. A major challenge lies in optimizing PBMT parameters such as wavelength (600–1,100 nm), energy density, session duration, and frequency, as existing studies vary significantly in these aspects. Systematic research and well-designed clinical trials are essential to establish protocols that ensure therapeutic efficacy without adverse effects. Combining PBMT with other treatments—such as corticosteroid injections to inhibit fibroblast proliferation or platelet-rich plasma (PRP) to promote tissue regeneration—may offer synergistic benefits (74), though the optimal sequence, dose, and frequency for such combinations remain to be defined.

In-depth molecular and genetic research is also critical to understanding PBMT’s role in keloid pathogenesis. Studies focusing on its effects on TGF-β signaling, collagen synthesis, and pro-fibrotic pathways such as Smad and PI3K/Akt could support the integration of PBMT with targeted molecular therapies (75). A personalized treatment approach is necessary, as individual responses to PBMT may vary based on ethnicity, keloid characteristics, age, and genetic factors. Moreover, intercompartmental signaling—particularly interactions between keratinocytes, fibroblasts, and immune cells—may influence keloid formation, though this remains poorly understood. In individuals with darker skin, melanocyte-derived factors like endothelin-1 and α-MSH may stimulate keratinocyte proliferation, potentially affecting PBMT outcomes (76). Addressing these factors through personalized protocols and interdisciplinary collaboration will be key to advancing PBMT as a standardized, effective therapy for keloid management.

In addition to PBMT, pharmacological agents that influence vascular function—such as statins—may warrant investigation. Statins, known for their vasodilatory and anti-inflammatory properties through nitric oxide modulation and endothelial stabilization, could hypothetically impact keloid vascularization and wound healing (77). However, the potential benefits must be weighed against possible long-term effects on skin integrity and aging, as statins have been associated with altered collagen turnover and reduced dermal thickness in some contexts. Future studies should explore these aspects to determine whether statins play a supportive or detrimental role in dermal remodeling.

Additionally, differences in vitamin D synthesis among skin phototypes may influence keloid susceptibility. Individuals with darker skin have lower efficiency in synthesizing vitamin D due to reduced UVB-mediated cutaneous conversion (78, 79). Given vitamin D’s role in regulating immune responses, fibroblast activity, and TGF-β1 expression, its deficiency might contribute to heightened keloid formation (80). Thus, activated (hydroxyated) vitamin D should be further investigated as a possible adjunctive therapy for individuals with dark skin prone to keloid development.

Furthermore, ultrastructural studies of the extracellular matrix (ECM)—using modalities like transmission electron microscopy (TEM) or second harmonic generation (SHG) imaging—could provide crucial insights into the microarchitectural changes in collagen organization pre- and post-PBMT. Keloids are characterized by dense, disorganized collagen fibers; detecting structural improvements over the course of treatment could validate PBMT’s long-term remodeling effects (81). These findings may also hold significance for broader matrix biology research, particularly in understanding fibrotic mechanisms across different tissues.

## Conclusion

Photobiomodulation therapy (PBMT) is a promising, non-invasive treatment for keloid management, acting through mitochondrial stimulation to enhance ATP production, reduce oxidative stress, and modulate fibroblast activity. It exhibits anti-fibrotic, immunomodulatory, and anti-angiogenic effects, supported by both preclinical and clinical studies showing reduced keloid size, symptoms, and recurrence with minimal side effects. While further research is needed to standardize treatment protocols and optimize dosing parameters, PBMT holds strong potential as a safe and effective alternative or adjunct to conventional therapies, particularly when integrated into combination regimens with agents like corticosteroids, retinoids, or platelet-rich plasma.

Importantly, consideration of individual factors—such as skin phototype and vitamin D status—may improve treatment outcomes and help explain the higher prevalence of keloids in darker-skinned populations. Additionally, ultrastructural evaluation of extracellular matrix remodeling may serve as a valuable biomarker of long-term efficacy, offering insights not only for scar modulation but also for broader fibrotic conditions. These perspectives underscore PBMT’s expanding relevance in both dermatological and regenerative medicine contexts.
